# Malarial Retinopathy in Bangladeshi Adults

**DOI:** 10.4269/ajtmh.2011.10-0205

**Published:** 2011-01-05

**Authors:** Abdullah Abu Sayeed, Richard J. Maude, Mahtab Uddin Hasan, Noor Mohammed, M. Gofranul Hoque, Arjen M. Dondorp, M. Abul Faiz

**Affiliations:** Chittagong Medical College Hospital, Chittagong, Bangladesh; Mahidol–Oxford Tropical Medicine Research Unit, Faculty of Tropical Medicine, Mahidol University, Bangkok, Thailand; Centre for Tropical Medicine, Centre for Clinical Vaccinology and Tropical Medicine, Churchill Hospital, Oxford, United Kingdom; Sir Salimullah Medical College, Mitford, Dhaka, Bangladesh

## Abstract

To establish if assessment of malarial retinopathy in adult malaria using ophthalmoscopy by non-ophthalmologists has clinical and prognostic significance, 210 Bangladeshi adults were assessed by both direct and indirect ophthalmoscopy; 20 of 20 healthy subjects and 20 of 20 patients with vivax malaria showed no retinal changes, whereas in patients with falciparum malaria, indirect ophthalmoscopy revealed malarial retinopathy (predominantly retinal hemorrhages) in 18 of 21 (86%) fatal, 31 of 75 (41%) cerebral, 16 of 64 (25%) non-cerebral but severe, and 1 of 31 (3%) uncomplicated cases. Direct ophthalmoscopy missed retinopathy in one of these cases and found fewer retinal hemorrhages (mean difference = 3.09; 95% confidence interval = 1.50–4.68; *P* < 0.0001). Severity of retinopathy increased with severity of disease (*P* for trend < 0.0001), and renal failure, acidosis, and moderate/severe retinopathy were independent predictors of mortality by both ophthalmoscopic techniques. Direct ophthalmoscopy by non-ophthalmologists is an important clinical tool to aid diagnosis and prognosis in adults with severe malaria, and indirect ophthalmoscopy by non-ophthalmologists, although more sensitive, provides minimal additional prognostic information.

## Introduction

Every year, more than 1 million people die of severe and cerebral malaria.^1^ Microvascular sequestration of infected erythrocytes is pivotal in the pathophysiology of the disease but difficult to assess in the living patient. An exception is the directly accessible retinal microvasculature, which is very similar to the cerebral microvasculature.^2^ Retinal changes observed in patients with severe malaria can, thus, mirror changes in the brain and be of pathophysiological, prognostic, and diagnostic significance.^2^ The occurrence of retinal hemorrhages in severe malaria has been known for over 130 years,^3^ but the unique spectrum of retinal signs in African children with cerebral malaria was first described in Malawi in 1993.^4^ This spectrum consists of four components: retinal whitening (macular or peripheral), vessel discoloration (white or orange), retinal hemorrhages (particularly with white centers), and papilloedema.^5^ Vessel discoloration and the pattern of retinal whitening are unique to and retinal hemorrhages are relatively specific for severe malaria, and they can be used for diagnostic purposes in high transmission areas in sub-Saharan Africa, where it is difficult to distinguish severe malaria from other causes of severe febrile illness presenting with incidental peripheral blood parasitaemia.^5^ Severity of malarial retinopathy in children also has important prognostic significance for both prolonged coma and death.^6^ Studies of ocular findings in adults have been far less extensive and hampered by the use of mostly direct ophthalmoscopy, which limits examination of the peripheral retina compared with indirect ophthalmoscopy. A recent study in Bangladesh using a portable retinal camera found the prevalence of malarial retinopathy in adults with cerebral malaria to be similar to that in children.^7^ The current study was undertaken to evaluate and compare direct and indirect ophthalmoscopy as bedside tools to aid diagnosis in adult patients with malaria admitted to a tertiary referral hospital in Chittagong, Bangladesh and to evaluate their prognostic utility.

## Materials and methods

### Study site and patients.

This observational study was conducted at Chittagong Medical College Hospital, a large, 1,000-bed teaching hospital in Chittagong, Bangladesh from May 2007 to December 2007.

Consecutive adult patients (> 16 years) with peripheral blood slide- or rapid diagnostic test (Paracheck, Orchid Biomedical Systems, Goa, India)-confirmed *Plasmodium falciparum* malaria were recruited if written informed consent was obtained from them or an attending relative. These patients were divided into the following groups: cerebral malaria (Glasgow Coma Score < 11 and no other obvious cause for coma, including hypoglycemia and post-ictal state), severe non-cerebral malaria (criteria adapted from table 3 in Tran and others^8^), uncomplicated *P. falciparum* malaria, and *P. vivax* malaria. Healthy volunteers were also recruited from the local population as a control group for comparison with the background prevalence of retinal changes in the healthy population. Exclusion criteria were patients unable or unwilling to cooperate with eye examination, contraindications to tropicamide and phenylephrine eye drops (angle closure glaucoma or documented allergy), severe corneal scarring or cataracts in both eyes precluding ophthalmoscopy, diabetes mellitus, hypertension, intracranial space occupying lesion(s), epilepsy, alcoholism, head injury, and chronic renal failure.

### Study procedures.

On admission, a full history and examination were carried out. Blood samples were obtained for hemoglobin, hematocrit, parasitemia, platelet count, leukocyte count, serum glucose, and full biochemistry. Eye examination included pupillary reaction to light and direct and indirect ophthalmoscopy. Because indirect ophthalmoscopy is more sensitive with a wider field of view and is much better for examining the peripheral retina (where many of the changes of malarial retinopathy are found), it was used as the gold standard and was always performed after direct ophthalmoscopy to minimize bias. Observations were made by two independent observers who underwent 2 weeks of intensive training to supplement their limited prior experience of direct and indirect ophthalmoscopy. In case of non-matching findings, the patient was reexamined, and findings were scored by consensus between the observers. Ophthalmoscopy was performed on admission, and in those who had changes on day 1, it was repeated 3 days after admission and at discharge. Two drops of tropicamide 0.5% or 1% eye drops, with the addition of 2.5% phenylephrine, were used for mydriasis. Retinal changes were graded as mild or moderate to severe by simplified criteria based on the classification of Beare and others^9^ and Harding and others^10^ (Table 1). A subgroup of unselected patients underwent retinal photography with a Zeiss Visucam Lite digital fundus camera (Carl Zeiss Ophthalmic Systems, Jena, Germany) when sufficiently well to be transported to the nearby Bangladesh National Society for the Blind Eye Hospital.

### Drug and supportive treatments.

Antimalarial medications and supportive treatments were in accordance with the 2006 World Health Organization (WHO) guidelines^11^ and local hospital guidelines, but the availability of renal replacement therapy and mechanical ventilation was limited.

### Statistical analysis.

Statistical analysis was carried out using the SPSS version 16.0 and Stata version 10. Correlation of malaria severity with retinopathy severity was by *P* value for trend. Numbers of retinal hemorrhages were compared using independent samples *t* test and Kruskal–Wallis test as appropriate. All the tests were two-sided, and *P* < 0.05 was considered to be statistically significant. Independent predictors of mortality were identified using a stepwise logistic regression procedure. Only variables with a *P* value of less than 0.05 were retained in the final model.

## Results

A total of 210 subjects were studied, of whom 75 had cerebral malaria, 64 had severe malaria but no coma, 31 had uncomplicated falciparum malaria, 20 had vivax malaria and 20 subjects served as healthy controls (Table 2).

The distribution of presenting severity features is shown in Table 3.

### Retinal findings.

**Figure 1.**
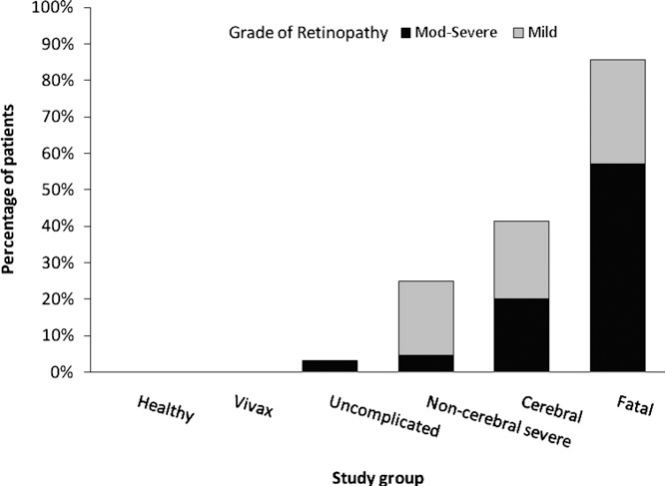
Severity of retinal changes consistent with malarial retinopathy in patients with *P. falciparum* or *P. vivax* malaria and healthy volunteers.

Features compatible with malarial retinopathy were found in 47 of 139 patients (34%) with severe malaria, 18 of 21 patients (86%) with a fatal course, 31 of 75 patients (41%) with cerebral malaria, 16 of 64 patients (25%) with non-cerebral severe malaria, 1 of 31 patients (3%) with uncomplicated malaria, 0 of 20 patients (0%) with *P. vivax* malaria, and 0 of 20 healthy volunteers (0%; *P* for trend < 0.0001) (Figure 1). The prevalences of individual features of retinopathy, as seen by indirect ophthalmoscopy, are shown in Table 4. The number of retinal hemorrhages seen on admission correlated with admission Glasgow Coma Score (*r*^2^ = 0.120; *P* < 0.0001).

### Direct versus indirect ophthalmoscopy.

A comparison of findings by direct and indirect ophthalmoscopy is shown in Table 5. There was no significant difference in the overall prevalence of retinopathy as assessed by these two techniques, but peripheral abnormalities were better detected by indirect ophthalmoscopy. With indirect ophthalmoscopy as reference, direct ophthalmoscopy had a high sensitivity to detect retinal hemorrhages and papilloedema but was less sensitive to detect retinal whitening (Table 4). More retinal hemorrhages were found by indirect versus direct ophthalmoscopy (mean difference = 3.09; 95% confidence interval [CI] = 1.50–4.68; *P* < 0.0001). In all but 10 patients, the overall severity of retinopathy was the same by direct and indirect ophthalmoscopy. In seven patients (3%), retinopathy was scored as severe by indirect ophthalmoscopy and mild by direct ophthalmoscopy, and in one patient, retinopathy was scored as mild by indirect ophthalmoscopy and absent by direct ophthalmoscopy; this was because of differences in the numbers of retinal hemorrhages seen. In three patients (1%), papilloedema was seen by direct ophthalmoscopy but not by indirect ophthalmoscopy.

### Disease outcome.

Indirect ophthalmoscopy findings in patients with fatal and non-fatal severe malaria are shown in Table 6. A logistic regression model using admission Glasgow Coma Score, overall severity of retinopathy, clinical renal failure (anuria or oliguria [< 20 mL/hour urine output]), clinical acidosis (Kussmal breathing), blood hemoglobin, hematocrit, plasma lactate, plasma glucose, blood urea nitrogen, serum creatinine, serum bilirubin, serum bicarbonate, and peripheral blood parasite count as independent variables (model 1 in Table 7) identified renal failure, acidosis, and moderate/severe retinopathy by either direct or indirect ophthalmoscopy as independent predictors for death. When the number of retinal hemorrhages and presence or absence of papilloedema were used instead of overall severity of retinopathy (model 2 in Table 7), renal failure, acidosis, and number of retinal hemorrhages were predictors of death for indirect ophthalmoscopy, and renal failure, acidosis, and admission Glasgow Coma Score were predictors of death for direct ophthalmoscopy. The mean number of hemorrhages in fatal cases of severe malaria was significantly higher than in non-fatal cases (mean = 9.0, 95% CI = 5.7–12.2 versus mean = 1.6, 95% CI = 1.0–2.2; *P* < 0.0001 for indirect ophthalmoscopy; mean = 4.2, 95% CI = 3.0–5.4 versus mean = 1.3, 95% CI = 0.9–1.8; *P* < 0.0001 for direct ophthalmoscopy). Of the patients with papilloedema, 8 of 9 (89%) also had other retinal findings (7 of 9 [78%] had hemorrhages and 2 of 9 [22%] had whitening), 7 of 9 (78%) of which were moderate to severe.

### Retinal photography.

**Figure 2.**
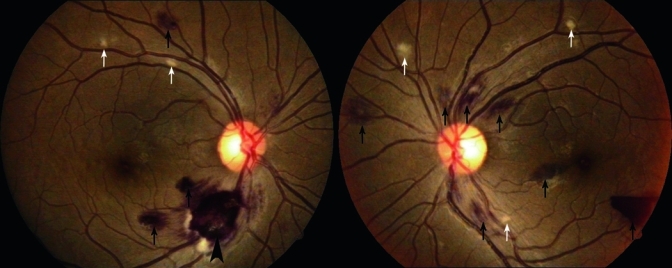
Fundus photographs of a patient with severe malaria showing a large white-centered hemorrhage (big black arrow), scattered patches of retinal whitening (white arrows), and hemorrhages (black arrows) in the right eye and multiple hemorrhages and patches of retinal whitening in the left eye.

Sixteen unselected patients with severe malaria underwent retinal photography when sufficiently well, and two examples are shown in Figure 2. The mean time after admission that this photography was performed was 4.8 days (95% CI = 3.6–5.9 days). In most patients, images were centered on the optic disc and fovea with a field of view of 45°, thus limiting capture of the peripheral retina. The retinal photographs were examined by a second blinded observer. Numbers of retinal hemorrhages on the photographs were similar to those observed by indirect ophthalmoscopy (mean difference = 1.1; 95% CI = −0.9–3.1; *P* = 0.252). Retinal whitening was found in 6 of 16 patients, and no vessel changes were seen. In only two of these patients was the retinal whitening found by indirect ophthalmoscopy, and in one of these patients, it was found by direct ophthalmoscopy. Both of these patients had marked changes in and around the macula. Papilloedema was not observed on the photographs.

### Follow-up.

The mean duration of admission for patients with malaria was 5.5 days (95% CI = 5.2–5.9 days); 42 patients with malaria and retinal changes on admission (25% of the total) were reexamined on day 3, and 30 of these were also examined at discharge. By day 3, the grade of retinal changes had improved in 1 of 42 patients (2%; this patient had retinal hemorrhages) and worsened in 0 patients. By the time of discharge, malarial retinopathy had fully cleared in 11 of 30 patients (37%), had improved in 2 of 30 patients (7%), and was the same as on admission in 17 of 30 patients (57%) who were assessed.

## Discussion

This study showed that over one-third of adult patients with severe malaria have malarial retinopathy on admission when assessed by non-expert direct and indirect ophthalmoscopy. Retinal hemorrhages are the most easily visible manifestation of malaria in the retina and as such, have the greatest potential as a bedside diagnostic and prognostic sign. This has been proposed for the diagnosis of pediatric cerebral malaria in high transmission settings in sub-Saharan Africa.^5^ In these settings, a positive peripheral blood slide for malaria does not necessarily imply that a child has clinical malaria because of the high background slide positivity rates. This was supported by a large prospective autopsy study of children with fatal cerebral malaria in Malawi, where it was found that malarial retinopathy was better than any other clinical or laboratory feature in distinguishing malarial from non-malarial coma.^12^ In low transmission settings like Bangladesh, the clinical diagnosis of severe malaria is not confounded by asymptomatic peripheral blood parasitemia, and the contribution of ophthalmoscopy to diagnosis is more limited.

The current study compared bedside direct and indirect ophthalmoscopy for the assessment of malaria retinopathy. There was little advantage gained from indirect ophthalmoscopy by a non-expert compared with direct ophthalmoscopy regarding overall sensitivity to detect malarial retinopathy. However, it is likely that, with increased training, indirect ophthalmoscopy would be more sensitive. In the current study, both techniques had similar prognostic value for a fatal course of the disease, suggesting that simpler direct ophthalmoscopy is an adequate tool to assess disease severity, although this was based predominantly on numbers of retinal hemorrhages alone. Both methods found significantly higher numbers of hemorrhages in fatal versus non-fatal infections. This was despite the number of retinal hemorrhages seen by indirect ophthalmoscopy being around double those seen by direct ophthalmoscopy.

The present study used a simplified version of the classification of malarial retinopathy used previously in children, which allowed direct comparison of findings across age groups and settings. The prevalences of retinopathy in the present study are around one-half of those detected by objective expert assessment using retinal photography in a smaller study in the same patient population^7^ and indirect ophthalmoscopy by experienced ophthalmologists in Malawian children.^6^ The present findings match prevalence rates found in previous studies in adults with malaria using ophthalmoscopy as a bedside tool. In a study by Kochar and others^13^ using a combination of direct and indirect ophthalmoscopy, retinopathy was present in 34% of patients with cerebral malaria, 24% of patients with severe non-cerebral malaria, and 1% of patients with uncomplicated falciparum malaria. Studies by Looareesuwan and others^14^ and Davis and others^15^ using direct ophthalmoscopy reported the prevalence of retinopathy in patients with cerebral malaria as 14.6% and 28%, respectively, predominantly as retinal hemorrhages. These discrepancies suggest that the sensitivity of non-expert direct or indirect ophthalmoscopy for detecting malarial retinopathy is likely to be around 50%.

The highly specific and common (around 50% of adults)^7^ retinal whitening and rarer vessel discoloration seen in previous studies^6^,^7^ were rarely (whitening) or never (discoloration) seen in the present study. This is true of most previous studies in adults^13^–^15^ and early studies in children,^16^,^17^ which predominantly described retinal hemorrhages.^5^ Retinal whitening frequently occurs without hemorrhages and can be difficult to see, particularly when it is in the peripheral retina,^18^ and in our experience, we have found that only the most severe whitening is easily visible by direct ophthalmoscopy and only after specific training of the physician. One-half of those with whitening on retinal photography in the present study were not detected by either ophthalmoscopy technique. Thus, it is likely that the incidence and severity of retinopathy in these patients were an underestimate. Further in-depth training and extended practice in indirect ophthalmoscopy may help to minimize this in future studies. Despite this shortcoming, the present study confirms the utility of assessment by direct ophthalmoscope of malarial retinopathy, especially retinal hemorrhages, as a simple bedside indicator of prognosis in this patient group. Because expert ophthalmological examination of severe malaria in the field setting is unlikely, the results in this study are a realistic reflection of what can be achieved in a real world setting.

The prognostic significance of retinal hemorrhages was independent of other well-established prognostic indicators, such as metabolic acidosis and renal failure, and is in accordance with previous studies (Thailand^14^ and Malawi^6^). This independence from other markers may be a reflection of the peculiar similarity between the retinal and cerebral vasculatures and the consequent pathology resulting from selective sequestration of parasites at these sites.^2^ The studies in Malawi have shown that the presence of malaria retinopathy, which includes not only hemorrhages but also retinal whitening, vessel changes, and papilloedema, is highly specific for a neuropathological diagnosis of cerebral malaria and, as assessed by an experienced ophthalmologist, is a much better predictor than hemorrhages alone; thus, this is recommended for the clinical diagnosis of pediatric cerebral malaria.^6^ The number of retinal hemorrhages on admission in the present study correlated with the depth of coma at the same time point. A previous study in Vietnam found no association between the presence of retinal hemorrhages and coma, but the study size was small.^19^ In studies in Kenyan and Malawian children, coma and retinal hemorrhages did correlate.^6^,^20^ It has also been shown in Malawian children with cerebral malaria that the presence of retinal hemorrhages during life correlates strongly with the presence of post-mortal cerebral hemorrhages at autopsy.^21^ These associations could indicate a pathogenetic relationship between hemorrhages and coma; alternatively, this could be incidental, because both hemorrhages and coma are likely to be the result of microcirculatory blockage.^2^ An autopsy study in Vietnamese adults has shown that the extent of sequestration of parasitized erythrocytes in the brain microvasculature correlates with the presence of coma,^22^ but other factors are also likely to contribute to the pathogenesis of coma.^2^

The frequency of papilloedema in this study was similar to the findings in the African studies, reporting a prevalence of 15% in pediatric cerebral malaria compared with 4% in children with severe malaria anemia.^6^ Papilloedema is caused by raised intracranial pressure, which, in patients with cerebral malaria, has been hypothesized to be mainly caused by the increase of the intravascular blood volume caused by the presence of the sequestered biomass.^23^ Increased intracranial pressure can also be caused by cerebral edema, and there is still considerable debate as to what extent cerebral edema contributes to coma in falciparum malaria. There is a mild generalized increase in the systemic vascular permeability in severe malaria, but the blood–brain barrier (BBB) in adults with cerebral malaria is functionally grossly intact^24^; however, in African children with cerebral malaria, there is a subtle increase in BBB permeability with a disruption of endothelial intercellular tight junctions on autopsy.^25^ Imaging studies reveal that most adults with cerebral malaria have no evidence of cerebral edema^26^ In African children, cerebral edema is more frequent, although not a consistent finding. Similarly, opening pressures on lumbar puncture are usually normal in adult patients but are elevated in over 80% of children with cerebral malaria.^27^ Use of mannitol to lower intracranial pressure in Ugandan children with cerebral malaria did not affect clinical outcome.^28^ In the present study, papilloedema was not an independent predictor of mortality.

In children, macular and peripheral whitening are frequent findings, being present in 44% and 46%, respectively, of those with cerebral malaria in one Malawian study.^6^ These studies also revealed that areas of retinal whitening coincide with non-perfused areas on fluorescein angiography^29^ and obstructed retinal capillaries on autopsy,^30^,^31^ strongly suggesting an ischemic etiology for these lesions.^2^,^29^ This is thought to mirror similar, usually reversible, patchy ischemia in the brain.^2^,^32^ A recent study in Bangladesh using a portable retinal camera found that the severity of retinal whitening correlated with severity of malaria and blood lactate levels, suggesting ischemia.^7^ The prevalence of peripheral whitening was much lower in the present study than previously in the same population using retinal photography,^7^ likely because of the much lower sensitivity of the techniques when used by a non-ophthalmologist.

No patients in this study had visible vessel discoloration. This includes those who underwent retinal photography. This finding agrees with the previous study in Bangladesh that used retinal photography.^7^ However, vessel discoloration in children is quite common^6^ and is thought to be caused by dehemoglobinization of sequestered erythrocytes by malaria parasites.^30^

Just under two-thirds of adult patients with malarial retinopathy in this study still had retinopathy at the time of discharge, and most retinal abnormalities did not improve during the admission. In Malawian children, it was shown that malaria retinopathy takes from 1 to 4 weeks to resolve. Additional studies in adults will be needed to carefully assess the dynamics of retinal pathology over time. This is particularly important for patients readmitted with a febrile illness after an episode with severe malaria to determine whether findings could persist from this previous episode.

Limitations of this study include its dependence on the skill of the examining doctor, because ophthalmoscopy is operator-dependent and thus, subjective. Study subjects were examined by non-ophthalmologists with relatively limited training in ophthalmoscopy. However, this should provide a realistic picture of what might be achieved by similarly skilled non-specialists at the bedside. In the 16 patients who underwent retinal photography, the findings closely matched those recorded by indirect ophthalmoscopy, with the exception that much of the retinal whitening seen on the photographs was missed by indirect ophthalmoscopy and papilloedema was overdiagnosed by direct ophthalmoscopy. In addition, direct and indirect ophthalmoscopy was performed by the same individuals on each patient, thus potentially biasing the second examination to match the first. For this reason, less sensitive direct ophthalmoscopy was always performed first.

In conclusion, non-expert direct ophthalmoscopy is an important prognostic tool in adults with severe malaria in the Bangladeshi setting. There is little additional information to be gained from non-expert indirect ophthalmoscopy in this setting.

## Figures and Tables

**Table 1:** Summary of simplified malarial retinopathy grading criteria

Feature	Grade of retinopathy	
	Mild	Moderate to severe
Whitening		
Macular	< 1/3 optic disc area	> 1/3 optic disc area(s)
Peripheral*	Occasional spots	More than occasional spots or patches of mosaic/confluence
Vessel changes*†		
Size of affected area	Small	Large
Number of vessel branches	Few	Many
Vessel length	Short	Long
Hemorrhages	1–5 in ≥ 1 eye	> 5 in ≥ 1 eye
Papilloedema	Absent	Present

* Sum of grades for each quadrant of the retina divided by number of quadrants seen.

† White or orange discoloration of capillaries and/or arterioles.

**Table 2:** Summary of patients recruited (*N* = 210)

Variable	Cerebral	Non-cerebral severe	Uncomplicated *P. falciparum*	*P. vivax*	Healthy
Number	75	64	31	20	20
Age					
Mean (years)	31.9	30.1	32.3	38.1	39.1
95% CI	29.0–34.8	26.9–33.3	26.6–37.9	30.8–45.3	35.2–42.9
Male (%)	75	63	65	75	50

**Table 3:** Distribution of presenting severity features in 139 patients with severe malaria

	Number of patients	
	Cerebral (*n* = 75)	Non-cerebral (*n* = 64)
Glasgow Coma Scale < 11	75 (100%)	0 (0%)
Severe anemia; hematocrit < 20%, or Hb < 5 mg/dL	12 (16%)	11 (17%)
Clinical jaundice	38 (51%)	56 (88%)
Renal failure; history of anuria or oliguria (i.e. < 20 mL/hour urine output)	19 (23%)	5 (8%)
Shock; systolic blood pressure < 80 mmHg with cool extremities	0 (0%)	0 (0%)
Peripheral asexual stage parasitemia > 10%	4 (5%)	0 (0%)
Venous lactate > 4 mmol/L	13 (17%)	5 (8%)
Acidosis; venous bicarbonate < 15 mmol/L	7 (9%)	4 (6%)

By definition, patients with uncomplicated malaria had none of these features.

**Table 4:** Prevalences of individual features of retinopathy in 170 patients with *P. falciparum* malaria as assessed by indirect ophthalmoscopy

Group	Retinal findings in *P. falciparum* malaria (indirect ophthalmoscopy; *n* = 170)						
	Any retinopathy	Hemorrhages		Papilloedema*	Whitening		
		White-centered	Any		Macular	Peripheral	Any
Cerebral (*n* = 75)							
Mild	16 (21%)	28 (37%)	20 (27%)		0 (0%)	1 (1%)	0 (0%)
Moderate-severe	15 (20%)	1 (1%)	9 (12%)	9 (12%)	4 (5%)	1 (1%)	4 (5%)
Total	31 (41%)	29 (39%)	29 (39%)	9 (12%)	4 (5%)	2 (3%)	4 (5%)
Non-cerebral (*n* = 64)							
Mild	13 (20%)	16 (25%)	13 (20%)		0 (0%)	0 (0%)	0 (0%)
Moderate-severe	3 (5%)	0 (0%)	3 (5%)	0 (0%)	0 (0%)	0 (0%)	0 (0%)
Total	16 (25%)	16 (25%)	16 (25%)	0 (0%)	0 (0%)	0 (0%)	0 (0%)
Uncomplicated (*n* = 31)							
Mild	0 (0%)	1 (3%)	0 (0%)		0 (0%)	0 (0%)	0 (0%)
Moderate-severe	1 (3%)	0 (0%)	1 (3%)	0 (0%)	0 (0%)	0 (0%)	0 (0%)
Total	1 (3%)	1 (3%)	1 (3%)	0 (0%)	0 (0%)	0 (0%)	0 (0%)

* All papilloedema was graded as moderate to severe. Vessel changes are not shown, because none were seen in this study. There were no abnormal findings in patients with *P. vivax* malaria or healthy volunteers therefore they are not shown.

**Table 5:** Comparison of findings in 170 patients with *P. falciparum* malaria by direct and indirect ophthalmoscopy

	Retinal findings in *P. falciparum* malaria (*n* = 170)						
	Any retinopathy	Hemorrhages		Papilloedema	Whitening		
		White-centered	Any		Macular	Peripheral	Any
Indirect	48 (28%)	46 (27%)	46 (27%)	9 (5%)	4 (2%)	2 (1%)	4 (2%)
Direct	47 (28%)	45 (26%)	45 (26%)	12 (7%)	3 (2%)	0 (0%)	3 (2%)
Sensitivity	94	98	98	100	75	0	75
Specificity	100	100	100	98	100	100	100

Vessel discoloration is not shown, because none was seen. Sensitivities and specificities are for direct ophthalmoscopy, using indirect ophthalmoscopy as the reference test.

**Table 6:** Indirect ophthalmoscopy findings in fatal and non-fatal severe malaria

Severity of retinopathy	Retinal findings in severe malaria (indirect ophthalmoscopy; *n* = 139)						
	Any retinopathy	Hemorrhages		Papilloedema*	Whitening		
		White-centered	Any		Macular	Peripheral	Any
Fatal (*n* = 21)							
Mild	6 (29%)	15 (71%)	8 (38%)		0 (0%)	0 (0%)	0 (0%)
Moderate to severe	12 (57%)	0 (0%)	8 (38%)	9 (43%)	2 (10%)	1 (5%)	2 (10%)
Total	18 (86%)	15 (71%)	16 (76%)	9 (43%)	2 (10%)	1 (5%)	2 (10%)
Non-fatal (*n* = 118)							
Mild	23 (19%)	29 (25%)	25 (21%)		0 (0%)	1 (1%)	0 (0%)
Moderate to severe	6 (5%)	0 (0%)	4 (3%)	0 (0%)	2 (2%)	0 (0%)	2 (2%)
Total	29 (25%)	29 (25%)	29 (25%)	0 (0%)	2 (2%)	1 (1%)	2 (2%)

* All papilloedema was graded as moderate to severe.

**Table 7:** Results of multiple logistic regression models for predicting death in patients with severe malaria

	Odds ratio for death (95% CI)	
	Indirect	Direct
Model 1		
Renal failure	32.1 (6.4–162.2), *P* < 0.0001	40.4 (8.0–204.3), *P* < 0.0001
Acidosis	14.3 (2.0–101.7), *P* = 0.008	6.4 (0.8–52.1), *P* = 0.081
Moderate to severe retinopathy	27.8 (4.4–177), *P* < 0.0001	41.9 (5.4–324.4), *P* < 0.0001
Model 2		
Renal failure	31.2 (6.6–148.0), *P* < 0.0001	37.6 (8.2–172.3), *P* < 0.0001
Acidosis	16.9 (2.8–102.2), *P* = 0.002	13.4 (2.2–80.4), *P* = 0.07
Number of retinal hemorrhages	1.2 (1.1–1.4), *P* = 0.004	
Admission Glasgow Coma Score		0.8 (0.6–0.9), *P* = 0.004
